# Integrative transcriptomics and structure-based screening identifies *Phyllanthus amarus* phytocompounds as potential WNT5A modulators in ovarian cancer

**DOI:** 10.3389/fbinf.2026.1815548

**Published:** 2026-05-21

**Authors:** Shreya Shibu, Abisha Sharon, Sidharth Kumar Nanda Kumar, Vasundra Vasudevan, Tarsha Muthukumar, Madhana Priya Nanda Kumar, D. Thirumal Kumar, R. Magesh

**Affiliations:** 1 Department of Biotechnology, Faculty of Biomedical Sciences & Technology, Sri Ramachandra Institute of Higher Education and Research (DU), Chennai, Tamil Nadu, India; 2 Meenakshi Academy of Higher Education and Research, Chennai, Tamil Nadu, India

**Keywords:** differential gene expression, GC–MS, healthcare, molecular docking, molecular dynamics, ovarian cancer, *P.amarus*, *WNT5A*

## Abstract

**Introduction:**

Ovarian cancer is one of the most lethal gynecological malignancies, largely due to its asymptomatic onset, heterogeneous molecular landscape, and frequent late-stage diagnosis. Identifying key oncogenic drivers and novel therapeutic candidates is important for understanding disease progression and guiding future research efforts.

**Methods:**

An integrative computational pipeline was employed to identify differentially expressed genes prioritize a candidate target, and screen plant compounds for anticancer potential. Transcriptomic datasets (GSE14407, GSE18520, and GSE26712) were retrieved from Gene Expression Omnibus and analyzed using GEO2R. The three-dimensional structure of *WNT5A* was obtained from AlphaFold. GC–MS profiling of *Phyllanthus amarus* Schumach. and Thonn. Identified phytocompounds for virtual screening, followed by molecular docking, ADME, and toxicity prediction. Molecular dynamics simulations, Free Energy Landscape and Dynamic Cross-Correlation Matrix analyses were performed. Further, Free energy perturbation (FEP) calculations were performed to estimate relative binding free energies across alchemical λ states.

**Results:**

A total of 103 consistently upregulated genes were identified, among which *WNT5A* emerged as a key regulator associated with tumor progression, metastasis, and chemoresistance. Enrichment analysis indicated involvement in mesenchymal development, extracellular matrix organization, and receptor-mediated signaling. GC–MS identified 48 compounds, and docking revealed several ligands with favorable interactions with *WNT5A*. MD simulations supported the structural stability of selected complexes, while FEL and DCCM analyses indicated stable conformational states and ligand-influenced residue dynamics. The FEP-derived free energy estimates further supported the stability and favorable binding of selected ligands toward WNT5A. These results enhance confidence in the computational predictions and complement the MD and docking analyses.

**Discussion:**

This integrative *in silico* framework highlights *WNT5A* as a potential therapeutic target and identifies bioactive compounds from *P. amarus* as candidates for further investigation. These findings provide preliminary computational insights and warrant experimental validation to improve treatment outcomes in healthcare settings.

## 1 Introduction

Ovarian cancer is the eighth most common cancer among women at the global level. Most cases are diagnosed at the advanced stages due to poor prognosis with patients presenting symptoms such as abdominal pain or pelvic pain, etc., prior to ovarian cancer diagnosis (Caruso et al., 2025). Ovarian cancer is a complex disease characterized by multiple underlying mechanisms and represents a significant global health concern (Zhang et al., 2021). This contributes to the higher mortality rate due to late stage detection with the symptoms being non-specific. Ovarian cancer originates from three types of cells namely-epithelial cell, germ cell and stromal cell (Reid et al., 2017).

Epithelial ovarian cancers are the most common (about 90%) and are categorised into five subtypes based on their histology. High-grade serous carcinomas (HGSC), Low-grade serous carcinomas (LGSC), Endometrioid carcinoma (EOC), Clear Cell Carcinoma of the Ovary (CCCO) and Mucinous Ovarian Carcinoma (MOC). Based on morphology and molecular alterations, epithelial ovarian cancers are categorised into two; type I and type II (Kurman and Shih, 2008). Type I consists of low grade, genetically stable tumors. These tumors are indolent and are associated with KRAS, BRAF, PTEN, and β-catenin. Type II tumors are more aggressive and unstable while being typically associated with p53 mutations. HGSC’s (Type II) are often associated with BRCA1 and BRCA2 mutations (Craig et al., 2023).

High grade serous carcinomas account for most of the ovarian cancers diagnosed at advanced stages (Stage III and IV). Therefore, early detection of HGSCs is an important research focus. It was previously believed to have originated from the ovaries; however, recent studies suggest that they might have emerged from the distal fallopian tube as STIC, that is, Serous Tubal Intraepithelial carcinoma presenting as the precursor lesion. However, not all STICs account for HGSCs and other potential molecular origins need to be investigated (Kim et al., 2018). LGSC (type I) is a rare subtype comparatively and can arise from an SBT; Serous Borderline Tumor (about 60%). Despite generally being diagnosed at the advanced stages, this type of serous tumor shows an improved survivability rate compared to HGSCs (Babaier et al., 2022).

Clear cell ovarian carcinomas (CCCO) have lower presentations of BRCA1 and BRCA2 mutations with wild type p53 (Kigawa et al., 2015). Significant mutations associated with CCCO include ARID1A, PIK3CA, TERT including TP53. CCCO are typically diagnosed at a younger age and about 50% of these cases including endometrioid carcinomas are associated with endometriosis. Diagnosis of clear cell carcinomas at an early age has a more favorable outcome compared to the advanced stages due to poor response to platinum based therapy (Iida et al., 2021). Several studies have revealed that PIK3R1 mutations along with PTEN or KRAS mutations are associated with endometriosis-associated ovarian cancer classified as typeI tumor (Rzepecka et al., 2025). In addition, PIK3R1 allele loss and decreased mRNA expression are associated with aggressive progression in tumors particularly typeII along with molecular alterations including PIK3R1 amplification, PTEN loss and p53 mutations (Driva et al., 2023).

Mucinous tumors could be benign, borderline or malignant which is further classified as invasive or non-invasive. These tumors present as neoplasms with unidentified molecular origin (Ricci et al., 2018). Hence, it continues to pose a significant threat to public health. According to data from the International Agency for Research on Cancer (IARC, 2024), there were about 3,294,316 new cases of breast, cervical and ovarian cancer in 2022 accounting for 16.5% of all cancer diagnoses worldwide (Bray et al., 2024). Among these malignancies, ovarian cancer is particularly concerning due to its non-specific symptoms and frequent diagnosis at an advanced stage.

In ovarian cancer, therapeutic management takes a multidisciplinary approach which includes debulking surgery, chemotherapy and rarely radiation therapy with a recent interest in using immunomodulation therapy. Despite available interventions, relapse rates remain high, approximately 25% for early-stage cancer and up to 80% for advanced stages (Nishino et al., 2010; Oh and Chari, 2019). Further treatments after the relapse are intensive, increasing the toxicity and resistance. One approach to reduce negative effects involves priming the cancer cells for subsequent treatments to work. Platinum-based chemotherapy drugs, such as cisplatin and carboplatin, have shown significantly improved responses after relapse, especially when cancer cells have been appropriately pre-treated. These drugs work by damaging cancer cell DNA through cross-linking, which ultimately triggers apoptosis (programmed cell death) (Dasari and Tchounwou, 2014). Despite these advances cancer remains a formidable challenge as it has involved mechanisms to resist treatment like reduced drug uptake, increased detoxification, or improved DNA repair. Moreover, the harsh side effects of these drugs can sometimes lead to the termination of treatment. Hence, the main goal is to identify effective drug targets that can halt cancer progression and enhance treatment outcomes, making therapies more successful and less burdensome for patients.​

Due to the complex and heterogeneous nature of ovarian cancer, identifying robust molecular targets remains challenging. Traditional approaches often focus on predefined candidates, which may overlook novel regulators. Therefore, unbiased, data-driven strategies using integrative transcriptomic analysis are essential for the systematic identification of key genes involved in disease progression.

Wnt signaling is categorised into two pathways based on β-catenin dependency, i.e., the canonical pathway which is dependent on β-catenin and the non-canonical pathway which is β-catenin independent. Several studies have confirmed that Wnt5a is responsible for the Epithelial-to-Mesenchymal transition (EMT) associated with cancer progression through a Ca^2+^ dependent pathway (Qi et al., 2014). Previous studies state that *WNT5A* can inhibit dysregulated β-catenin activation that leads to cell proliferation and resistance to tumors in ovarian cancer cells through the activation of the non-canonical pathway leading to tumor suppression. However, its interaction with the ROR2 receptor promotes Rac1 inducing cell invasion and migration in ovarian tumors (Grither et al., 2024). In this context, the study employs an integrative, data-driven approach to identify key genes associated with ovarian cancer progression.

*Phyllanthus amarus* Schumach. and Thonn. Is a well-known medicinal plant with a broad spectrum of pharmacological properties, including anticancer, antioxidant, and anti-inflammatory activities (Patel et al., 2011). The plant is rich in diverse bioactive phytochemicals such as lignans, flavonoids, alkaloids, and phenolic compounds, which have been reported to exhibit potential therapeutic effects in various disease conditions. Owing to its established medicinal relevance and chemical diversity, *Phyllanthus amarus* was selected in the study as a source of natural compounds for exploratory screening. This selection was not based on prior evidence of direct interaction with *WNT5A* but aimed to identify potential bioactive molecules through a computational, target-based approach.

This study aims to identify key oncogenic drivers linked with ovarian cancer through integrative transcriptomic analysis and to prioritize possible therapeutic targets. Through cross-dataset transcriptomic analysis and network-based prioritization, *WNT5A* emerged as a prominent candidate gene, which was subsequently selected for further investigation. This approach ensures an unbiased identification of potential targets and provides a basis for downstream computational analyses. Additionally, the study tries to evaluate phytocompounds from *P. amarus* for their potential interaction with the selected target using computational methods, such as molecular docking and molecular dynamics simulations. Overall, this work is intended to provide preliminary insights into target identification and compound screening for future experimental validation.

## 2 Methodology

### 2.1 Dataset retrieval

Transcriptomic datasets relevant to ovarian cancer were retrieved from the NCBI Gene Expression Omnibus (GEO) (Edgar et al., 2002). The search criteria included: *Homo sapiens* datasets, availability of both normal and cancer samples, and adequate sample size for robust differential gene expression analysis. Three datasets GSE14407, GSE18520, and GSE26712 were selected. To ensure compatibility, metadata, sample platforms, and series matrices were examined. Only primary tumor datasets were included in order to avoid technical bias and all datasets were obtained in raw format for reproducible downstream analysis.

### 2.2 Differential gene expression analysis using GEO2R

DEG identification was performed using the GEO2R (Barrett et al., 2013) tool, through the limma package executing linear modeling and empirical Bayes analysis effectively finding significantly expressed genes by improving the signal-to-noise ratio. Groups were defined manually for each dataset into Normal and Cancer groups. The p-value <0.05 and log2FC ≥ 1 were adjusted and used as thresholds. The gene expression data were log2-transformed and normalized using the default settings present in GEO2R. Multiple testing correction was conducted using the Benjamini–Hochberg method to reduce the false discovery rate. Probe-to-gene mapping was performed using the corresponding GEO platform annotation files. From the three datasets, the gene lists were exported and Venny 2.0 (Oliveros, 2007–2015) was utilised for identifying common DEGs. It is a web-based tool that creates interactive Venn diagrams and helps to compare about four to five datasets. Common upregulated genes from the datasets were prioritized as consistent overexpression indicates potential biological relevance in ovarian cancer progression (Chaudhary et al., 2026).

### 2.3 Plotting and visualization

Volcano plots were generated to visualize the distribution of significant DEGs. A volcano plot is a type of scatter plot that is used to identify statistically significant, highly upregulated and downregulated features. SRplot tool (Tang et al., 2023) was utilised to perform GO enrichment analysis in three categories, namely, Biological Process (BP), Cellular Component (CC), and Molecular Function (MF). This enables classification of genes based on their function, biological roles and cellular locations Cnetplots generated mapped the significant pathways to the corresponding genes and the potential pathways related to ovarian cancer tumorigenesis were emphasised.

### 2.4 Stage-dependent expression and clinical implications of WNT5A in ovarian cancer

Expression analysis of WNT5A across different stages of ovarian cancer was performed using the UALCAN Tool (Chandrashekar et al., 2022). Gene expression levels were quantified as transcripts per million (TPM) and compared among Stage I–IV samples to assess stage-specific variation. Box plot visualization was used to represent the distribution and median expression across stages. For clinical relevance, survival analysis was conducted using the Kaplan–Meier Plotter database (Lánczky and Győrffy, 2021). Patients were stratified into high- and low-expression groups based on the auto-selected optimal cutoff (percentile-based). Progression-free survival (PFS) was selected as the clinical endpoint, and survival differences between groups were evaluated using the log-rank test. Hazard ratios (HR) with corresponding confidence intervals were calculated to determine the prognostic significance of WNT5A expression in ovarian cancer (Omoboyede et al., 2025).

### 2.5 Protein retrieval and processing

FASTA sequences and functional annotations for *WNT5A* were obtained from the UniProt database (Ahmad et al., 2025). It is a high quality, comprehensive resource for accessing protein sequence and functional information. PyMOL (Seeliger and de Groot, 2010), a molecular visualization program, was used to examine the AlphaFold model with the highest confidence that was obtained from the AlphaFoldDB. In order to find the stable conformation for molecular docking and MD simulations, the structure was optimized by eliminating water molecules and assigning the proper protonation states. This step was followed by Energy Minimization using the Swiss-PDB viewer which is crucial for achieving stable conformations and to refine molecular geometries. The predicted protein structure was validated using multiple computational tools. The stereochemical quality was assessed using a Ramachandran plot (PROCHECK) (Tam et al., 2023). Overall model quality was evaluated using ProSA-web (Wiederstein and Sippl, 2007) to determine the Z-score. Verify-3D (Rani et al., 2025) was used to check the compatibility of the 3D structure with its amino acid sequence, while ERRAT (Binbay et al., 2023) analyzed non-bonded interactions to assess structural reliability. Additionally, energy profiling methods were employed to examine local model stability. These validation steps ensured the suitability of the model for further analyses (Kumari and Dalal, 2022).

### 2.6 Phytochemical extraction

Fresh leaves of *P. amarus* were collected from Kolathur, Chennai, India. The leaves that were harvested were shade-dried, powdered finely and using 100% ethanol as solvent, it was subjected to Soxhlet extraction. About 10 g of the powdered plant material was wrapped using filter paper and this was further extracted with 400–500 mL of ethanol for about 6–8 h until the siphon tube became clear (colourless) which indicates complete extraction. The extract that was obtained was then concentrated using a rotary shaker under pressure at 40 °C–45 °C and this was further dried to obtain a crude ethanolic extract.

### 2.7 GC-MS analysis of the crude extract

Gas Chromatography–Mass Spectrometry (GC–MS) profiling of the ethanolic extract was performed using a Thermo Scientific TRACE 1300 gas chromatograph coupled with a Thermo Scientific Integrated Single Quadrupole Detector (ISQ QD) mass spectrometer. All standard analytical conditions recommended for plant metabolite analysis were followed. Briefly, 1 µL of the filtered extract was injected into a DB-5 MS column (30 m × 0.25 mm × 0.25 µm) in split mode. The oven temperature program was initiated at 60 °C and gradually increased to 280 °C at a rate of 3 °C/min. To ensure data quality and reproducibility, solvent blank injections were performed to monitor carryover, and the instrument was routinely tuned and calibrated prior to analysis. Compound identification was carried out by comparing the obtained mass spectra with entries in the NIST library database.

### 2.8 Ligand retrieval

The phytochemical constituents of *P. amarus* identified through GC–MS analysis were retrieved from the PubChem database (Kim et al., 2025). These 2D structures were further converted into 3D format using openbabel (O’Boyle et al., 2011) and their smiles were also recorded for ensuring reproducibility and for future reference. These ligands were subjected to initial preparation including energy minimization using Swiss pdb viewer (Guex et al., 2009) and saved as PDBQT files. To ensure compatibility with docking workflow, the compounds lacking structural information, ambiguous stereochemistry, missing atoms, were excluded from further analysis and studies.

### 2.9 Virtual screening

The Python Prescription (Pyrx) tool (Dallakyan and Olson, 2015) was used for structure-based virtual screening and Prankweb tool (Jendele et al., 2019) was used for the prediction of the binding region in the *WNT5A* gene along with the functional residues as it provides clear information on surface cavities and functional amino acid residues which are crucial for understanding interaction regions within the WNT5A protein. A grid box was defined enclosing the probable ligand-binding region. The grid dimensions were adjusted to cover the active site while avoiding unnecessary conformational space. Docking was performed for all 48 phytochemicals and the binding affinities were sorted. Shortlisting was based on potential compounds with higher binding affinity, stable interaction profiles and favourable orientation with the active site. These shortlisted compounds were taken into consideration for further computational and dynamic analyses to determine and validate their therapeutic potential.

### 2.10 ADME and toxicity prediction

SwissADME (Daina et al., 2017) and protox 3 (Banerjee et al., 2024) were used for evaluating pharmacokinetic properties and toxicity of the compounds. These are very essential in early stages of drug discovery as they contribute to reduced experimental cost and time. Specific parameters were checked for toxicity assessment including Lipinski’s rule compliance, solubility, GI absorption, blood–brain barrier permeability and hepatotoxicity. Protox was used to estimate hepatotoxicity, mutagenicity and cytotoxicity risks along with their LD 50 values and their toxicity classes (Priya Nanda Kumar et al., 2025). Compounds with acceptable drug-likeness profiles were selected and the final filtered set contained compounds with balanced efficacy and toxicity profiles which was proceeded further for molecular docking interpretation and dynamic studies (Ranjani et al., 2026).

### 2.11 Molecular docking

Molecular docking of phytocompounds with *WNT5A* was carried out using AutoDock 4 (Morris et al., 2009). The grid box was centered at coordinates x- 0.398, y- 1.706, z- 5.712 with dimensions of 126 × 120 × 126 grid points and a spacing of 0.4638 Å. The docking grid was defined based on the predicted binding/active regions of the protein, ensuring appropriate coverage of functionally relevant residues. Due to the lack of a well-defined ligand-bound crystal structure for WNT5A, the binding site was predicted using the PrankWeb server (P2Rank), which identifies ligand-binding pockets based on structural and physicochemical features. Receptor structure preparation involved assigning Kollman charges, removing water molecules and adding polar hydrogen bonds. All ligands were geometrically optimized along with Gasteiger charges assignment, and converted into PDBQT format. Appropriate atomic charges were assigned using AutoDock Tools (ADT4), including Kollman united atom charges, which were added to the protein to represent electrostatic properties of amino acid residues. This is essential to perform protein–ligand interaction calculations. The use of Kollman charges for proteins is a standard protocol in AutoDock and ensures accurate modeling of electrostatic interactions during docking simulations (Dalal et al., 2021). Identification of the binding pocket was done using Prankweb and AutoGrid was used for enclosing the probable active region of *WNT5A* using the grid box generated to optimize protein-ligand binding (Priya et al., 2026). The Lamarckian Genetic Algorithm (LGA) is utilised by docking simulations for robust sampling followed by selecting the lowest binding energy conformation for each ligand, and the interaction profiles were examined for bondings and residue arrangement using Discovery Studio (Madhana Priya et al., 2023).

### 2.12 Molecular dynamics and post MD analysis

The protein-ligand complexes selected after docking were subjected to 500 ns all-atom Molecular Dynamics (MD) simulations using GROMACS (Pronk et al., 2013) to evaluate their conformational stability and their dynamic behaviour. Each of the systems was solvated in a cubic simulation box with TIP3P water molecules and appropriate counter-ions were added to secure electrical neutrality. Energy minimization was carried out using the steepest descent algorithm to remove steric clashes and unfavorable interactions. The minimized systems were then sequentially equilibrated under NVT and NPT ensembles to stabilize the temperature at 300 K and pressure at 1 bar, respectively (Madhana Priya et al., 2024). Long-range electrostatic interactions were treated using the Particle Mesh Ewald (PME) method, and hydrogen bond constraints were maintained using the LINCS algorithm. Subsequently, a 500 ns production run was performed under periodic boundary conditions. Post-simulation analyses, including Root Mean Square Deviation (RMSD), Root Mean Square Fluctuation (RMSF), Radius of Gyration (Rg), hydrogen bond occupancy, and Principal Component Analysis (PCA), were also conducted to test the structural stability, conformational flexibility, and ligand retention within the binding pocket throughout the simulation period (Priyanka et al., 2025).

### 2.13 Free Energy Landscape (FEL) plot

Free Energy Landscape (FEL) analysis (Prada-Gracia et al., 2009) was performed to determine the conformational stability and energy minima of the protein–ligand complex during the molecular dynamics simulation. The FEL plots were constructed by using principal component analysis (PCA) that were derived from the MD trajectory. The first two principal components (PC1 and PC2), that represented the major collective motions of the system, were selected as reaction coordinates. The Gibbs free energy (ΔG) was calculated using the Boltzmann distribution equation, and the energy landscape was also visualized to identify stable conformational states and transition regions. Lower energy basins determined energetically favorable and stable conformations of the complex.

### 2.14 Dynamic Cross-Correlation Matrix plot

Dynamic Cross-Correlation Matrix (DCCM) analysis (Arnold and Ornstein, 1997; Prada-Gracia et al., 2009) was done to evaluate correlated and anti-correlated motions among amino acid residues throughout the MD simulation. The DCCM was generated from the C-alpha atom trajectories of the protein by using standard correlation algorithms. Positive correlation values indicated coordinated movement between residues, whereas negative values represented motion in the opposite direction. The analysis provided valuable insights on internal residue-residue communication, domain movements, and the influence of ligand binding to the protein dynamics. The correlation maps were further visualized using appropriate plotting tools for interpretation.

### 2.15 Free energy perturbation (FEP)

Alchemical free energy calculations (Mey et al., 2020) were performed using the Bennett Acceptance Ratio (BAR) method to estimate binding free energy differences between ligand-bound states. The alchemical transformation was carried out across a series of discrete λ windows (λ = 0 → 1), enabling a gradual conversion between initial and final states. At each λ value, molecular dynamics simulations were performed to adequately sample conformational space, and free energy changes (ΔG) were calculated from the ensemble averages. To improve convergence and reduce numerical instability, Coulombic (electrostatic) and van der Waals (Lennard–Jones) interactions were decoupled in a stepwise manner. The individual contributions from these interactions were analyzed separately, providing insights into their respective roles in stabilizing ligand–protein binding. The cumulative free energy change was obtained by integrating contributions across all λ windows, yielding a reliable estimate of the overall binding free energy.

## 3 Results

### 3.1 Dataset retrieval

Three ovarian cancer transcriptomic datasets (GSE14407, GSE18520 and GSE26712) were sourced from GEO, from 282 samples including 32 normal ovarian tissues and 250 primary ovarian tumor samples. The datasets were selected based on sample diversity, high - quality annotation, and balanced experimental design. The combined datasets provided sufficient statistical power for validation of differential gene expression profiles across various platforms. The samples from laser-microdissected tissues or whole - tumor biopsies were biologically relevant upon metadata inspection. After normalization, gene expression matrices were consistent across platforms allowing integrative comparative analysis. These datasets offered robust transcriptomic landscapes suitable for identifying clinically significant biomarkers such as *WNT5A* (Table 1).

**TABLE 1:** GEO datasets with accession ID, sample size and description retrieved from Gene Expression Omnibus Database.

GEO dataset	Sample size	Normal	Cancer
GSE14407	24	12	12
GSE18520	63	10	53
GSE26712	195	10	185

### 3.2 Differential gene expression analysis using GEO2R

**FIGURE 1:**
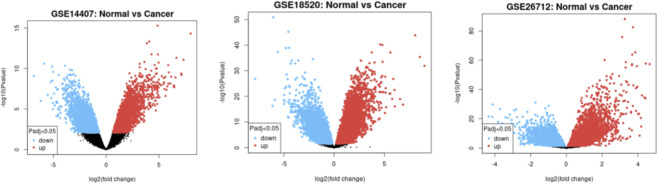
Volcano plot illustrating significantly up- and downregulated genes based on fold change and p-value thresholds

**FIGURE 2:**
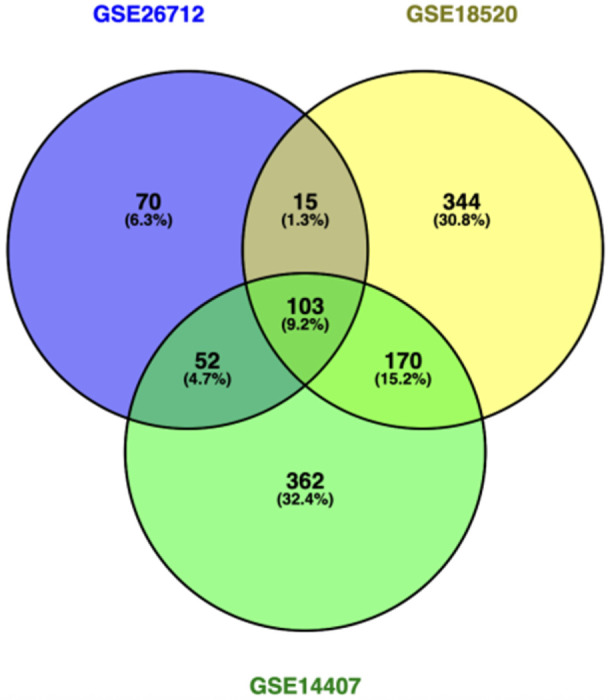
Venn diagram generated using Venny depicting unique and shared genes within the datasets.

Distinct sets of upregulated and downregulated genes for each dataset were released through GEO2R analysis with thousands of DEGs meeting the adjusted p-value threshold of <0.05. Clear segregation was presented between significantly upregulated and downregulated genes in the volcano plot (Figure 1), specifically in GSE18520 and GSE26712, exhibiting broader transcriptomic shifts. Venn analysis (Figure 2) identified 103 genes consistently overexpressed across all datasets from the comparison across the three datasets, presenting high-confidence molecular signatures associated with ovarian cancer progression. Several developmental regulators, signaling molecules, and extracellular matrix-related genes were among the most significantly upregulated and *WNT5A* emerged as one of the common consistently elevated genes reinforcing its potential role in ovarian tumorigenesis.

DEGs were identified for each GEO dataset with Padj<0.05. All three datasets showed a significantly clear separation of upregulated and downregulated genes. Most DEGs show high −log10 (p-values), especially in GSE18520 and GSE26712 datasets, indicating reliable and biologically meaningful gene expression differences.

The analysis performed using Venny software showed that there were 103 genes in common across all three datasets, indicating a strong consensus gene signature. Dataset-specific DEGs were also reflected, showing 70 genes unique to GSE26712, 344 genes unique to GSE18520, and 362 genes unique to GSE14407. Furthermore, partial overlaps were identified between the dataset pairs, which included 15 genes overlapped between GSE26712 and GSE18520, 52 genes between GSE26712 and GSE14407, and 170 genes between GSE18520 and GSE14407. These findings revealed that there were both shared and dataset-specific transcriptional variations, which suggests potential core biomarkers along with condition- or cohort-specific gene expression patterns.

**FIGURE 3:**
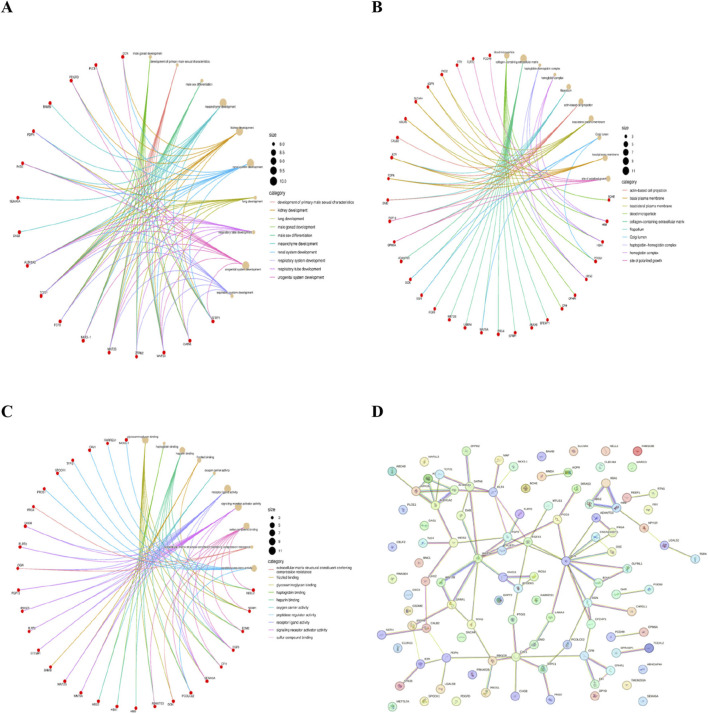
Gene Ontology (GO) Enrichment Analysis of Differentially Expressed Genes. **(A)**- BP cnetPLOT, **(B)**-CC cnetPLOT, **(C)**- MF cnetPLOT, **(D)**- PPI Network.

Significant functional alterations in ovarian cancer were observed in the GO enrichment analysis. Biological Process enrichment highlighted activation of pathways related to urogenital system development, mesenchyme development, extracellular matrix assembly, and tissue morphogenesis processes known to drive invasive behavior. kStrong enrichment in the collagen-containing extracellular matrix, Golgi lumen, and basolateral plasma membrane was seen in the Cellular Component analysis indicating ECM remodelling and vesicular secretion capable of supporting tumor growth. Molecular Function enrichment (Figure 3) presented overrepresentation of receptor ligand activity, glycosaminoglycan binding, and frizzled binding with these dysregulations aligning to the Wnt pathway. *WNT5A* was consistently mapped to multiple enriched pathways, confirming its functional relevance in ovarian cancer biology.

A protein–protein interaction (PPI) network was constructed by utilising the STRING database to find key hub genes from the common differentially expressed genes (DEGs). PPI networks represent a web of intricate molecular connections that provide crucial insights into cellular processes and disease mechanisms. Analysis of the network topology was performed to determine the significance of each node present within the network. The hub genes were ordered based on degree centrality, which represents the number of interactions a particular node has with other proteins present in the network. Additionally, betweenness centrality (representing role of node in the information flow) and closeness centrality (reflecting the average shortest route to other nodes) were also assessed to guarantee strong prioritisation. Those genes that showed higher values across the mentioned topological parameters were regarded as key hub genes. *WNT5A* was found to be a highly connected hub protein with strong functional interactions, underscoring its pivotal regulatory role in tumor progression.

### 3.3 Stage-dependent expression and clinical implications of WNT5A in ovarian cancer

**FIGURE 4:**
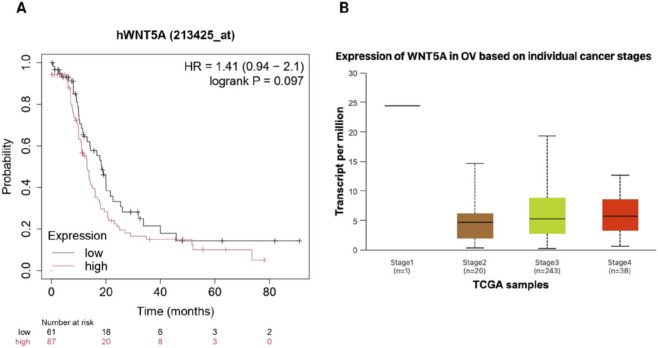
**(A)** Kaplan–Meier curve of overall survival based on WNT5A expression (low vs. high); higher expression shows a non-significant trend toward poorer survival (HR = 1.41, P = 0.097). **(B)** Box plot of WNT5A expression across cancer stages (TCGA), showing increased expression with advancing stage.

Stage-wise analysis showed that WNT5A is expressed across all ovarian cancer stages, with a trend toward increased expression in advanced stages (Stage III and IV). Stage II exhibited moderate expression, while Stage III and IV showed higher median values and greater variability, indicating heterogeneity in later stages and a possible role in disease progression. Kaplan–Meier survival analysis revealed that higher WNT5A expression was associated with reduced progression-free survival (HR = 1.41), suggesting an increased risk of disease progression (Figure 4). Overall, these results suggest that WNT5A may contribute to ovarian cancer progression and holds potential clinical relevance.

### 3.4 Protein and ligands structure retrieval

The three-dimensional structure of *WNT5A* was retrieved from the AlphaFold database (UniProt ID: P41221-2). The predicted model showed an average pLDDT score of 89.56 which indicates high confidence. Residue-level analysis showed that 79.2% of residues were in the very high confidence range, while smaller proportions were found in high (9%), low (5.8%), and very low (6%) confidence categories. We acknowledge that no comparison with experimentally determined structures was performed, due to the limited availability of *WNT5A* structures in the Protein Data Bank (PDB). Therefore, the AlphaFold model was used as the best available structural representation. The model exhibited a compact fold with defined secondary structural elements and functional regions similar to the Wnt family. Energy minimization removed steric clashes and improved structural feasibility for docking simulations (Table 2).

**TABLE 2:** Energy minimization report of the modeled *WNT5A* structure showing reduced energy demonstrating convergence towards stable conformation.

KJ/mol	328.693	1,137.218	1,463.355	276.779	−11392.69	−13284.21	0.0000//E = −21470.861

**FIGURE 5:**
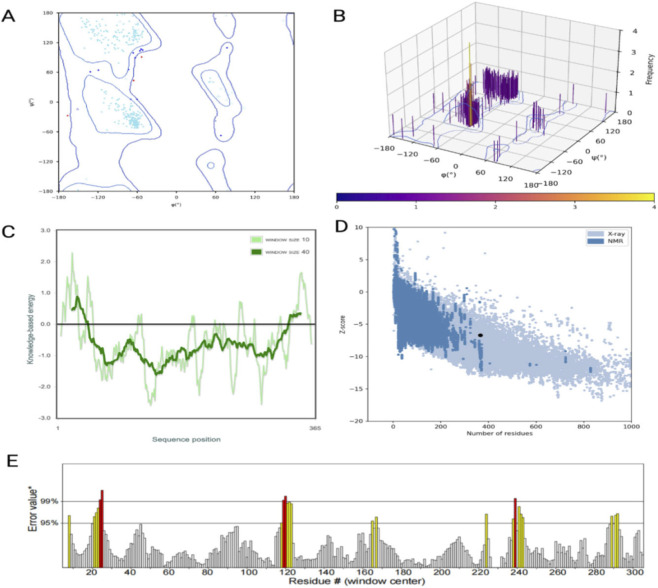
**(A)** Ramachandran plot showing the distribution of backbone dihedral angles (φ and ψ) of amino acid residues, with the majority located in favored and allowed regions. **(B)** Three-dimensional representation of residue distribution in the Ramachandran space, indicating conformational preferences. **(C)** Knowledge-based energy profile of the protein structure across sequence positions, demonstrating predominantly favorable energy values. **(D)** ProSA Z-score plot of the model compared with experimentally determined structures, confirming that the model falls within the acceptable range. **(E)** ERRAT analysis showing the overall quality factor based on non-bonded atomic interactions, with most regions exhibiting low error values, indicating good structural reliability.

The Ramachandran plot analysis indicated that the majority of amino acid residues were located within the most favored and additionally allowed regions, confirming acceptable stereochemical geometry of the model. The overall quality of the structure was further assessed using ProSA, where the Z-score of the model was found to be within the range of experimentally determined structures of similar size, indicating good model reliability. In addition, the local model quality was examined through knowledge-based energy profiling, which showed predominantly negative energy values across the sequence, suggesting a stable structure. The ERRAT analysis also demonstrated satisfactory non-bonded interaction quality, with most regions exhibiting low error values. Collectively, these validation results confirm that the predicted protein structure is reliable and suitable for molecular docking and subsequent computational analyses (Figure 5).

**FIGURE 6:**
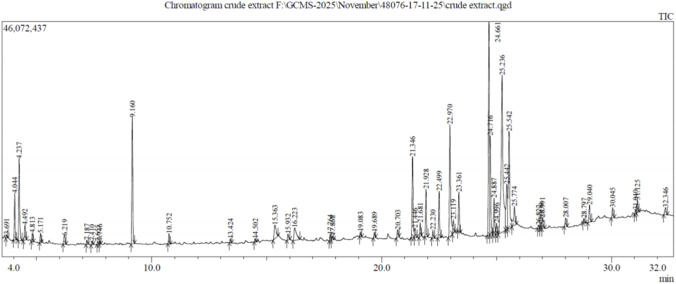
GC–MS chromatogram of crude extract showing multiple peaks at different retention times, indicating the presence of diverse phytochemical constituents.

Parallelly, the GC–MS analysis of the crude extract identified 48 phytoconstituents (Supplementary Table S1) which further revealed multiple peaks, confirming the presence of various bioactive compounds, with major peaks observed at specific retention times indicating dominant constituents (Figure 6), each structurally diverse in polarity, aromaticity, and chain length. A specialized library of natural compounds designed specifically for screening potential inhibitors of *WNT5A* was obtained.​

### 3.5 Virtual screening

Pyrx (Dallakyan and Olson, 2015) was used to analyze how each of the 48 phytochemicals might bind to *WNT5A* with a main focus on the predicted binding pockets of the protein and score them based on the predicted binding affinity and shape complementary. Various compounds showed favorable orientation within its predicted cavity, paving way for receptor modulation (Table 3). Screening helps identify compounds with initial binding energies and proper occupation of hydrophobic and partially buried residues in *WNT5A*. Compounds that fail to fit or exhibit poor scoring were eliminated, ensuring that the most suitable binders advanced to refined docking analysis.

**TABLE 3:** Binding affinities of the top 15 compounds extracted from *Phyllanthus amarus.*

Ligand	Binding affinity
wnt5a_emp_18274_uff_E = 330.48	−6.7
wnt5a_emp_135,398,635_uff_E = 534.89	−6.4
wnt5a_emp_80584_uff_E = 181.34	−5.6
wnt5a_emp_5325830_uff_E = 155.64	−5.5
wnt5a_emp_439250_uff_E = 101.93	−5.4
wnt5a_emp_439507_uff_E = 203.67	−5.4
wnt5a_emp_7463_uff_E = 91.91	−5.4
wnt5a_emp_21599893_uff_E = 113.59	−5.2
wnt5a_emp_567149_uff_E = 289.48	−5.1
wnt5a_emp_3026_uff_E = 212.61	−5
wnt5a_emp_5280934_uff_E = 142.21	−5
wnt5a_emp_5280435_uff_E = 165.89	−4.9
wnt5a_emp_5282184_uff_E = 2059.37	−4.9
wnt5a_emp_5366244_uff_E = 163.26	−4.9
wnt5a_emp_91693811_uff_E = 291.14	−4.9

### 3.6 ADME and toxicity prediction

ADME profiling was done through SWISS ADME (Bugnon et al., 2024) and toxicity prediction using Protox three was used to filter out ligands based on their pharmacokinetic and safety properties with a number of phytochemicals exhibiting high gastrointestinal absorption, favorable lipophilicity and non-violations of Lipinski’s rules supporting the potential oral-drug likeness. From the shortlisted compounds, Toxicity evaluation confirmed the absence of mutagenicity, hepatotoxicity and skin sensitization. Compounds exhibiting a high predicted clearance -which measures how quickly the drug is removed by the body via metabolism and excretion and low bioavailability were prioritized. Filtering ensured that only structurally promising and pharmacologically viable candidates were subjected to detailed docking and MD simulation. The candidate compounds include 2,7 -Dioxaspiro (4.4)nonane-1,6-dione, Phenylacetaldehyde diethyl acetal (−)-Limonene, D-Allose and Dibutyl phthalate.

### 3.7 Molecular docking

**FIGURE 7:**
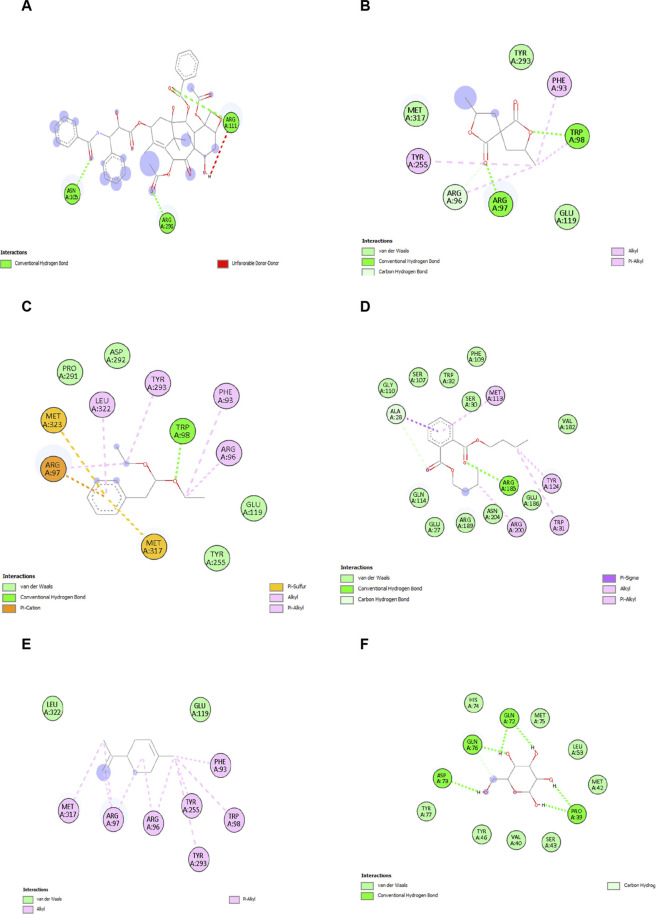
Molecular docking interaction results of the ligand–protein complex demonstrating hydrogen bonds and hydrophobic interactions within the active site. **(A)** Paclitaxel. **(B)** 2,7 -Dioxaspiro(4.4)nonane-1,6-dione. **(C)** Phenylacetaldehyde diethyl acetal. **(D)** Dibutyl phthalate. **(E)** (-)-Limonene. **(F)** D-Allose.

The compounds selected for molecular docking were based on their predicted pharmacokinetic properties (ADME) and low toxicity profiles which was evaluated using ProTox and ADME prediction tools, along with their potential relevance to cancer-associated pathways. The binding affinities obtained ranged from −4.9 to −6.7 kcal/mol, indicating moderate interactions and are considered as preliminary findings in the context of drug discovery. The binding interaction involved key amino acid residues such as GLY, LEU, GLN, ARG, and MET, which may contribute to the stability and specificity of the protein–ligand interaction. No RMSD-based validation of docking poses or benchmarking with known Wnt pathway ligands was performed, which represents a limitation of the study. Therefore, the docking results are interpreted as initial screening insights, requiring further validation through advanced computational and experimental studies. Several *P. amarus* compounds exhibited significant binding affinity towards *WNT5A* in the docking simulations done using AutoDock four compared to the control drug (Paclitaxel). Residues formed stable hydrogen bonds with these phytocompounds, indicating potential for interference with *WNT5A*–receptor interactions (Figure 7) Strong complementarity was demonstrated between these phytochemicals and the receptor cavity due to the hydrophobic contacts and π-alkyl interactions anchoring the ligand further into the binding pocket. Ligands presented repeated selective targeting of certain functional domains. Visual inspection supports the stability of the interactions. These outcomes collectively support the likelihood of *WNT5A* modulation by selected *P. amarus* compounds.

### 3.8 Molecular dynamics

**FIGURE 8:**
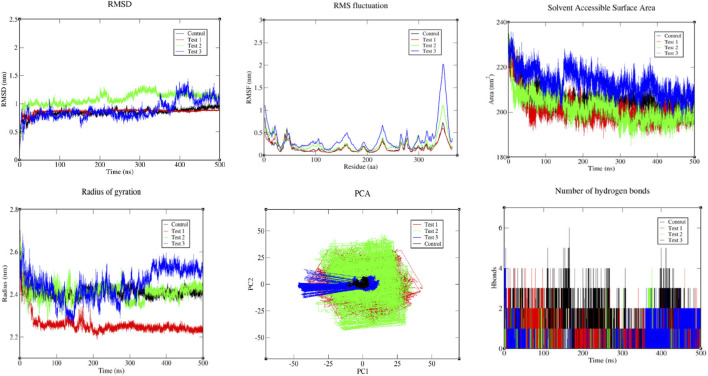
Molecular dynamics plot representing RMSD, RMSF, Solvent Accessible Surface Area, Radius of Gyration, PCA and Number of Hydrogen bonds of the control and test drugs with WNT5A.

The 500 ns molecular dynamics simulations for the Wnt5a protein with the Control Paclitaxel, Test 1–2,7 -Dioxaspiro (4.4)nonane-1,6-dione, Test 2- Phenylacetaldehyde diethyl acetal and Test 3- Dibutyl phthalate were performed to evaluate structural stability of the docked complexes. RMSD plot is used to visualise the stability of the complexes over a trajectory, based on the results, all three tests showed moderate stability with Test one presenting with the least fluctuations showing higher stability among the tests. The RMSF plot provides the real-time behaviour of the amino acid residues over a trajectory providing insights into its structural integrity. All three tests presented moderate fluctuations with Test three having greater fluctuations however, these did not exceed 2 Å indicating structural flexibility with conformational changes over the trajectory. The radius of gyration plot for the compactness of the protein over the 500 ns trajectory showed that among all the tests, Test one presented more compactness with relatively lesser fluctuations compared to Test2,Test three and control with higher magnitude of fluctuations. In addition, the SASA plot shows that Test 1,2 and control have lower values compared to Test three indicating more compact or stable conformations (Figure 8).

The MD simulation results indicate that all systems maintained overall structural stability, with comparable RMSD values for the control (0.849 ± 0.068) and Test 1 (0.863 ± 0.044), while Test 2 (1.088 ± 0.111) showed relatively higher deviation. Increased residue-level flexibility was observed in Test 2 (0.254 ± 0.179) and Test 3 (0.374 ± 0.316) based on RMSF. The radius of gyration remained largely stable, with Test 1 (2.260 ± 0.048) appearing slightly more compact and Test 3 (2.449 ± 0.069) slightly expanded. SASA values were generally consistent, though Test 3 (213.122 ± 5.599) indicated increased solvent exposure. Hydrogen bond analysis showed reduced interactions in ligand-bound systems, particularly in Test 2 (0.092 ± 0.385), compared to the control (1.040 ± 0.909). Overall, ligand binding preserved global stability while influencing flexibility, compactness, and interaction patterns (Table 4).

**TABLE 4:** Mean ± SD values of RMSD, RMSF, radius of gyration (Rg), SASA, and hydrogen bonds for control and ligand-bound systems (Test 1–3), indicating structural stability, flexibility, compactness, and interaction strength.

Parameter	Control (mean ± SD)	Test 1 (mean ± SD)	Test 2 (mean ± SD)	Test 3 (mean ± SD)
RMSD	0.849 ± 0.068	0.863 ± 0.044	1.088 ± 0.111	0.891 ± 0.158
RMSF	0.185 ± 0.131	0.176 ± 0.120	0.254 ± 0.179	0.374 ± 0.316
Radius of gyration	2.409 ± 0.023	2.260 ± 0.048	2.410 ± 0.036	2.449 ± 0.069
SASA	205.455 ± 5.540	201.022 ± 5.776	201.704 ± 5.919	213.122 ± 5.599
Hydrogen bonds	1.040 ± 0.909	0.367 ± 0.603	0.092 ± 0.385	0.373 ± 0.654

### 3.9 Free Energy Landscape (FEL) plot

**FIGURE 9:**
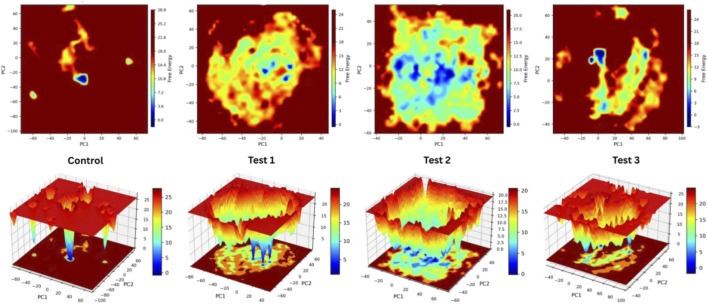
2D and 3D Free energy landscape plots (FEL) plots of the control, test 1, test 2 and test 3 with the protein, constructed using the first two principal components (PC1 and PC2) during molecular dynamics simulations.

The Free Energy Landscape analysis revealed that the *WNT5A* and ligand complexes demonstrated stability (Figure 9). Specifically, the control *WNT5A* and Paclitaxel complexes exhibited significant stability, maintaining a low-energy state consistently throughout the simulation. The *WNT5A* and Paclitaxel complex did not undergo substantial conformational changes, indicating its stability in this configuration thus serving as a good reference. The Free Energy Landscape analysis of ligand complexes is crucial for understanding their stability. The *WNT5A* and 2,7-Dioxaspiro (4.4)nonane-1,6-dione complexes, as depicted in Panel B, displayed multiple energy levels, suggesting potential conformational flexibility. This complex is not highly stable and may undergo conformational changes upon binding with other molecules. The *WNT5A* and Phenylacetaldehyde diethyl acetal complex, shown in Panel C, exhibited a distinct and deep energy basin with minimal variation, closely resembling the control complex and, in some aspects, surpassing it. This indicates that the *WNT5A* and Phenylacetaldehyde diethyl acetal complexes are highly stable and favorable, suggesting that the *WNT5A* and Phenylacetaldehyde diethyl acetal complex could serve as an effective *WNT5A* modulator, highlighting its significant potential. The *WNT5A*–dibutyl phthalate complex (Panel D) displayed two distinct low-energy basins, indicating the presence of multiple favorable conformational states and moderate stability, accompanied by conformational switching, which suggests adaptive binding dynamics without compromising the overall integrity of the complex.

### 3.10 Dynamic Cross-Correlation Matrix plot

**FIGURE 10:**
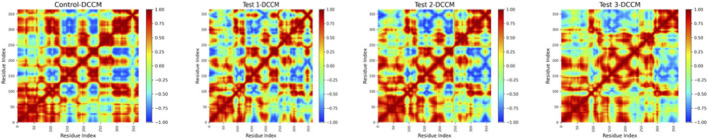
Dynamic Cross-Correlation Matrix analysis of protein-ligand complex showing residue-wise correlated motions for the control, test 1, test 2 and test three during molecular dynamics simulations.

The DCCM plot for the control and Tests 1, 2 and three exhibited correlated motions with these patterns presenting synchronised movements providing insights into the structural compactness and stability of the complexes (Figure 10). The Test complexes presented significant distribution of overall correlated motions and moderate reduction of anticorrelated motions compared to the plotted control. Among the test complexes, Test one and three presented a broader reduction of anti parallel motions compared to the control.

### 3.11 Free energy perturbation (FEP)

**FIGURE 11:**
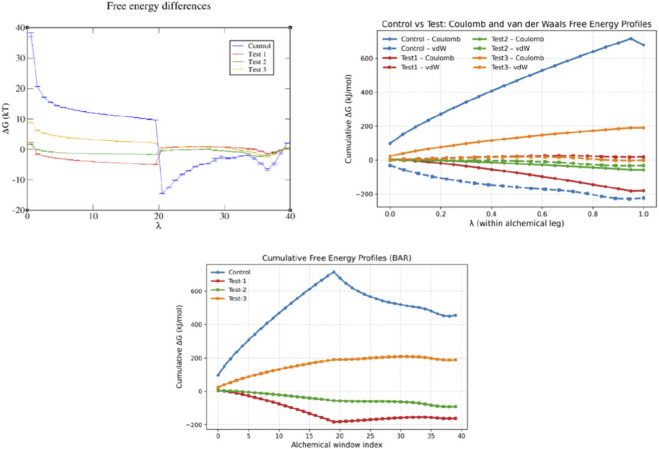
Alchemical free energy profiles of control and test compounds calculated using the BAR method. ΔG variation across λ windows, along with Coulombic and van der Waals contributions, are shown. The cumulative free energy profiles indicate improved binding affinity of the test compounds compared to the control.

Alchemical free energy calculations using the Bennett Acceptance Ratio (BAR) method provided detailed insights into the binding energetics of the ligand–protein complexes (Figure 11). Compared to the control, all test compounds exhibited lower ΔG values, indicating more favorable binding. Among them, Test-1 showed the most negative free energy, followed by Test-2, while Test-3 displayed moderate affinity. The cumulative free energy (ΔG) profiles across multiple λ windows exhibited smooth transitions with minimal fluctuations, indicating good convergence and sufficient sampling throughout the simulations. The stepwise decoupling of interactions revealed that both Coulombic and van der Waals components contributed to binding, with van der Waals interactions showing a relatively stronger influence on overall stability. The consistency of ΔG values across λ states suggests reliable estimation of binding affinities and stable ligand accommodation within the binding site. Collectively, these results align with the molecular docking and MD simulation findings, further confirming the stability and favorable interaction profiles of the selected ligands with WNT5A.

## 4 Discussion

Despite the presence of multiple interventions, advanced stages of ovarian cancer lack effective treatment strategies and remain the common cause of mortality among women. Hence identifying robust molecular targets and alternative therapeutic targets is essential. The study used an integrative computational framework combining both transcriptomic meta-analysis and structure-based virtual screening to identify the potential modulators of *WNT5A* in ovarian cancer. This dual level strategy was designed to improve target reliability while also simultaneously increasing early-stage drug discovery. By analyzing multiple gene expression datasets rather than focusing only on a single cohort, the study was able to reduce dataset-specific bias and also improved the robustness of differentially expressed gene (DEG) identification. The Venn-based approach helped in the recognition of consensus genes that are consistently dysregulated across independent studies, thereby increasing biological confidence in the selected molecular targets.

Functional analysis highlighted *WNT5A* as a prominent candidate gene, given its involvement in multiple pathways associated with cancer progression. *WNT5A* is an essential component of the noncanonical pathway and is expressed in many human tissues. It is crucial for the invasion, metabolism, and migration of human ovarian cancer. Studies have highlighted *WNT5A* messenger RNA overexpression in epithelial ovarian cancers compared to normal ovaries or benign epithelial tumors (Matei et al., 2002). With respect to this, previous studies have also highlighted the fact that upregulation of *WNT5A* leads to integrin modulation that is, it induces cell proliferation and cell invasion (Azimian-Zavareh et al., 2021; Matei et al., 2002). Current research aims to develop drugs targeting the *WNT5A* gene however, the molecular mechanisms related to the signaling pathway is yet to be fully understood. Several studies suggest the regulation of *WNT5A* in combination with chemotherapy. Recent studies have also highlighted higher expression of Wnt5a in Cancer Associated Fibroblasts (CAFs) playing a significant role in OC metastasis and maintenance of Ovarian cancer stem cells with high levels correlating with poor prognosis (Fang et al., 2024). Studies with SKOV3/Wnt5a with *WNT5A* overexpression and SKOV3/miRNA with Wnt5a downregulation revealed that Wnt5a overexpression decreased chemosensitivity meanwhile, its downregulation increased chemosensitivity. When compared to other well-established ovarian cancer biomarkers like TP53, BRCA1/2, and MUC16 (CA-125) (Radu et al., 2021), which relate to DNA repair mechanisms, genomic stability and also clinical diagnosis. *WNT5A* is a distinct biomarker that is involved in cell signaling pathways and non-canonical Wnt signaling pathway which regulates processes like cell migration, polarity and invasion. These functions suggest that *WNT5A* can have a more direct role in tumor aggressiveness and the advancement of metastasis compared to traditional markers. WNT5 is more than a potential tumor target, evidence shows that it plays an important role in ovarian cancer chemotherapy. This is supported by various studies that suggest the use of Wnt5a inhibitors along with the standard Ovarian cancer chemotherapy that may have the potential to reduce disease occurrence.

*P.amarus* is a therapeutic plant widely found in the tropical and subtropical regions having various phytocompounds such as saponins, flavonoids, triterpenes, etc., with significant therapeutic potential. Various studies have also demonstrated the cytotoxic effect of Phyllanthus amarus plant extracts on tumor cell lines of breast cancer and lung cancer (Lee et al., 2013). Opting *P. amarus* as a phytochemical source is well supported by its long-term ethnopharmacological activities and its pharmacological properties which includes antioxidant, anti-inflammatory, hepatoprotective, and anticancer activities (Patel et al., 2011). The present findings of the study extends this knowledge by showcasing a specific molecular mechanism *WNT5A* pathway modulation by which *P. amarus* derived compounds may show therapeutic potential in ovarian cancer. This mechanistic linkage also adds translational value by connecting traditional medicinal knowledge with modern computational drug discovery. This plant is capable of targeting the Wnt pathway which contributes to the progression of tumors. Therefore, this property of the Phyllanthus amarus extracts are highlighted and utilised in this study as a potential candidate for the treatment of EOC.

Further performing structure-based virtual screening of phytocompounds derived from *P. amarus* demonstrated several candidates that showed favorable binding affinities toward the *WNT5A* protein. The docking results revealed significantly negative binding energy values, indicating moderate ligand–protein interaction potential and also thermodynamic stability of the complexes. Interaction analysis was able to further highlight the presence of hydrogen bonds, hydrophobic contacts, and van der Waals interactions with critical amino acid residues within the predicted binding pocket. These interactions reflect structural complementarity and provide valuable instincts to the possibility of functional modulation or inhibition of *WNT5A* activity. The consistency found across docking scores and interaction patterns explains the reliability of the selected phytocompounds as promising lead molecules requiring further benchmarking and experimental studies. Energy minimization and refinement of conformation of the docked complexes further supported structural suitability as it reduced steric clashes and optimised bond geometries. The reduction in total potential energy after minimization shows increased molecular stability and also supports the plausibility of the predicted binding orientations. Such structural validation is very crucial, as energetically unfavorable conformations may lead to false-positive docking results. The combined docking and minimization studies gives a coherent structural motive for the expected bio-activity of the screened phytocompounds. Furthermore, molecular dynamics simulations and free-energy calculations provided deeper insights into binding stability over time under physiological conditions.

The identification of *P. amarus* phytocompounds as potential *WNT5A* modulators provides a promising layout for the development of plant-derived targeted therapeutics in ovarian cancer management., the convergence of multi-dataset transcriptomic evidence, molecular docking affinity, and structural stability analyses collectively suggests that selected *P. amarus* phytocompounds may serve as potential modulators of *WNT5A* signaling. These findings evidence further biochemical and pharmacological validation to demonstrate their efficacy, safety, and clinical applicability. The study also highlights the growing importance of integrative computational methodologies in oncology drug discovery and highlights the translational potential of natural product-based therapeutic exploration.

## 5 Limitations of the study

Despite the promising outcomes, the study is confined to its exclusive dependence on *in silico* methodologies. Computational docking and transcriptomic analyses provide valuable insights but cannot fully showcase and explain biological complexity, pharmacodynamics, or pharmacokinetic behavior in living models and systems. Experimental validation like *in-vitro* cell line assays, enzymatic inhibition assays, gene expression modulation studies, and *in-vivo* animal models are required to provide strong evidence to the predicted interactions and therapeutic relevance. Specifically, functional assays in ovarian cancer cell lines, including viability (e.g., MTT/CellTiter-Glo), migration and invasion assays, and evaluation of EMT and Wnt signaling markers (via qPCR/Western blot), would be valuable to confirm the modulation of *WNT5A*-mediated pathways by the identified compounds. Another important aspect is the heterogenetic nature of ovarian cancer, which has diverse histological subtypes and genetic backgrounds. Though Integrative transcriptomics reduces dataset-specific bias, it can still overlook subtype-specific molecular signatures. Future studies integrating gene expression with survival data will be essential to establish the prognostic significance and translational potential of *WNT5A*.

## 6 Conclusion

This study adopts an integrative computational strategy which combines transcriptomic analysis and structure-based virtual screening to find potential modulators of *WNT5A* in ovarian cancer. Using a multi-dataset transcriptomic analysis, *WNT5A* was found as a consistently upregulated and biologically relevant therapeutic target involved in ovarian cancer. Computational screening of 48 phytochemicals derived from *P. amarus* showed compounds with optimal predicted affinity and favorable pharmacokinetic properties. Molecular docking and MD simulations further confirmed the structural stability and interaction potential of top ligands with the *WNT5A* protein. The results provide a foundation for future validation through experiments and support the development of natural compound–based therapeutic ways targeting *WNT5A*-driven ovarian cancer progression. Overall, this study highlights the importance and effectiveness of combined bioinformatics and structure-based screening approaches in accelerating natural product-based drug discovery and offers valuable insights into *WNT5A*-targeted therapeutic development in ovarian cancer.

## Data Availability

The original contributions presented in the study are included in the article/Supplementary Material, further inquiries can be directed to the corresponding author.
