# Supplementary material for: Integrative transcriptomics and structure-based screening identifies Phyllanthus amarus phytocompounds as potential WNT5A modulators in ovarian cancer
Source: Front Bioinform. 2026 May 21;6:1815548. doi: 10.3389/fbinf.2026.1815548 (PMC13234468; doi:10.3389/fbinf.2026.1815548)
Supplement: Supplementary file 1 [file Table1.docx]

**Integrative Transcriptomics and Structure-Based Screening Identifies *Phyllanthus amarus* Phytocompounds as Potential WNT5A Modulators in Ovarian Cancer**

# **Shreya Shibu^1#^, Abisha Sharon^1#^, Sidharth Kumar N^1#^, Vasundra V^1^, Tarsha S M^1^, Madhana Priya N^1^, D Thirumal Kumar^2^ and Magesh R^1^***

^1^ Department of Biotechnology, Faculty of Biomedical Sciences & Technology, Sri Ramachandra Institute of Higher Education and Research (DU), Chennai.

^2^ Meenakshi Academy of Higher Education and Research, Chennai, Tamil Nadu, 600078, India.

# - Authors contributed equally

* Corresponding Author (Email: [magesh.r@sriramachandra.edu.in](mailto:magesh.r@sriramachandra.edu.in))

*Corresponding Author: Dr. Magesh Ramasamy, Department of Biotechnology, Sri Ramachandra Institute of Higher Education and Research (DU), Porur 600116, Chennai, Tamil Nadu, India. Email: [magesh.r@sriramachandra.edu.in](mailto:magesh.r@sriramachandra.edu.in)

**Supplementary Table 1: List of Phytochemicals with retention time in *Phyllanthus amarus***

| **Peak#** | **R.Time** | **Name** | **Canonical SMILES** |
| --- | --- | --- | --- |
| **1** | 3.691 | Propanoic acid, 2-oxo-, ethyl ester | CCOC(=O)C(=O)C |
| 2 | 4.044 | 1,1-DIETHOXY-2-BUTENE | CCOC(C=CC)OCC |
| 3 | 4.237 | 2-HYDROXY-2-METHYL-4- PENTANONE (DIACETONE) | CC(=O)CC(C)(C)O |
| 4 | 4.492 | PROPANE, 1,1-DIETHOXY-2- METHYL- | CCOC(C(C)C)OCC |
| 5 | 4.813 | Acetic acid, 3,3-dimethylbut-2-yl ester | CC(C(C)(C)C)OC(=O)C |
| 6 | 5.171 | 4-ETHOXY-2-PENTANONE | CCOC(C)CC(=O)C |
| 7 | 6.219 | Pentane, 1,1-diethoxy- | CCCCC(OCC)OCC |
| 8 | 7.187 | 3,8-DIMETHYL-2,7- DIOXASPIRO[4.4]NONANE-1,6- DIONE | CC1CC2(CC(OC2=O)C)C(=O)O1 |
| 9 | 7.410 | 1,1,3-TRIETHOXYBUTANE | CCOC(C)CC(OCC)OCC |
| 10 | 7.655 | BENZENE, 1-METHYL-4-(1- METHYLETHYL)- | CC1=C(C=C(C=C1)C(C)C)[N+](=O)[O-] |
| 11 | 7.746 | CYCLOHEXENE, 1-METHYL-4- (1-METHYLETHENYL)-, (S)- | CC1=CC[C@H](CC1)C(=C)C |
| 12 | 9.160 | 1,1,3-TRIETHOXYBUTANE | CCOC(C)CC(OCC)OCC |
| 13 | 10.752 | 3-Cyclohexen-1-ol, 4-methyl-1-(1- methylethyl)-, (R)- | CC1=CC[C@](CC1)(C(C)C)O |
| 14 | 13.424 | Benzene, (2,2-diethoxyethyl)- | CCOC(CC1=CC=CC=C1)OCC |
| 15 | 14.502 | 3-HEXADECENE, (Z)- | CCCCCCCCCCCC/C=C\CC |
| 16 | 15.363 | GUANOSINE | C1=NC2=C(N1[C@H]3[C@@H]([C @@H]([C@H](O3)CO)O)O)N=C(N C2=O)N |
| 17 | 15.932 | 1-Dodecanol | CCCCCCCCCCCCO |
| 18 | 16.223 | D-Allose | C([C@@H]1[C@H]([C@H]([C@H] (C(O1)O)O)O)O)O |
| 19 | 17.764 | 1-Hexadecanol | CCCCCCCCCCCCCCCCO |
| 20 | 17.868 | PENTADECANE | CCCCCCCCCCCCCCC |
| 21 | 19.083 | n-Tridecan-1-ol | CCCCCCCCCCCCCO |
| 22 | 19.689 | Ethanol, 2-(dodecyloxy)- | CCCCCCCCCCCCOCCO |
| 23 | 20.703 | 2-NONENAL, 2-PENTYL- | CCCCCC/C=C(/CCCCC)\C=O |
| 24 | 21.346 | Neophytadiene | CC(C)CCCC(C)CCCC(C)CCCC(=C)C=C |
| 25 | 21.446 | 4,8,12,16-Tetramethylheptadecan-4- olide | CC(C)CCCC(C)CCCC(C)CCCC1(C CC(=O)O1)C |
| 26 | 21.681 | 3,7,11,15-Tetramethyl-2-hexadecen- 1-ol | CC(C)CCCC(C)CCCC(C)CCC/C(= C/CO)/C |
| 27 | 21.928 | Neophytadiene | CC(C)CCCC(C)CCCC(C)CCCC(=C)C=C |
| 28 | 22.230 | 3,7,11-Trimethyl-2,4-dodecadiene | C/C=C(\C)/C=C/CC(C)CCCC(C)C |
| 29 | 22.499 | Hexadecanoic acid, methyl ester | CCCCCCCCCCCCCCCC(=O)OC |
| 30 | 22.970 | n-Hexadecanoic acid | CCCCCCCCCCCCCCCC(=O)O |
| 31 | 23.119 | Dibutyl phthalate | CCCCOC(=O)C1=CC=CC=C1C(=O)OCCCC |
| 32 | 23.361 | HEXADECANOIC ACID, ETHYL ESTER | CCCCCCCCCCCCCCCC(=O)OCC |
| 33 | 24.661 | 9,12-Octadecadienoic acid (Z,Z)-, methyl ester | CCCCC/C=C\C/C=C\CCCCCCCC(=O)OC |
| 34 | 24.716 | 11-Octadecenoic acid, methyl ester | CCCCCC/C=C/CCCCCCCCCC(=O) OC |
| 35 | 24.887 | Phytol | C[C@@H](CCC[C@@H](C)CCC/C (=C/CO)/C)CCCC(C)C |
| 36 | 24.996 | Methyl stearate | CCCCCCCCCCCCCCCCCC(=O)O C |
| 37 | 25.236 | 9,12,15-Octadecatrienoic acid, (Z,Z,Z)- | CC/C=C\C/C=C\C/C=C\CCCCCCC C(=O)O |
| 38 | 25.442 | 9,12-Octadecadienoic acid (Z,Z)- | CCCCC/C=C\C/C=C\CCCCCCCC(=O)O |
| 39 | 25.542 | ETHYL (9Z,12Z)-9,12- OCTADECADIENOATE # | CCCCC/C=C\C/C=C\CCCCCCCC(=O)OCC |
| 40 | 25.774 | OCTADECANOIC ACID, ETHYL ESTER | CCCCCCCCCCCCCCCCCC(=O)O CC |
| 41 | 26.822 | Octanoic acid, 2-dimethylaminoethyl ester | CCCCCCCC(=O)OCCN(C)C |
| 42 | 26.930 | Heneicosane | CCCCCCCCCCCCCCCCCCCCC |
| 43 | 26.991 | cis-11,14-Eicosadienoic acid, methyl ester | CCCCC/C=C\C/C=C\CCCCCCCCC C(=O)OC |
| 44 | 28.007 | Hexacosane | CCCCCCCCCCCCCCCCCCCCCC CCCC |
| 45 | 28.797 | 3-Cyclopentylpropionic acid, 2- dimethylaminoethyl ester | CN(C)CCOC(=O)CCC1CCCC1 |
| 46 | 29.040 | 2-Methylhexacosane | CCCCCCCCCCCCCCCCCCCCCC CCC(C)C |
| 47 | 30.045 | Tetratetracontane | CCCCCCCCCCCCCCCCCCCCCC CCCCCCCCCCCCCCCCCCCCCC |
| 48 | 31.040 | 9,12,15-Octadecatrienoic acid, (Z,Z,Z)- | CC/C=C\C/C=C\C/C=C\CCCCCCC C(=O)O |
